# SARS-CoV-2 variants of concern Alpha and Delta show increased viral load in saliva

**DOI:** 10.1101/2022.02.10.22270797

**Published:** 2022-02-15

**Authors:** Kylie L. King, Stevin Wilson, Justin M. Napolitano, Keegan J. Sell, Lior Rennert, Christopher L. Parkinson, Delphine Dean

**Affiliations:** 1 Center for Innovative Medical Devices and Sensors (REDDI Lab), Clemson University, Clemson, South Carolina, United States of America.; 2 Clemson University Genomics and Bioinformatics Facility, Clemson, South Carolina, United States of America.; 3 Department of Public Health Sciences, Clemson University, Clemson, South Carolina, United States of America.; 4 Department of Biological Sciences and Department of Forestry and Environmental Conservation, Clemson University, Clemson, South Carolina, United State of America; 5 Department of Bioengineering, Clemson University, Clemson, South Carolina, United States of America.

## Abstract

**Background:**

Higher viral loads in SARS-CoV-2 infections may be linked to more rapid spread of emerging Variants of Concern. Rapid detection and isolation of cases with highest viral loads, even in pre- or asymptomatic individuals, is essential for the mitigation of community outbreaks.

**Methods and Findings:**

In this study, we analyze Ct values from 1297 SARS-CoV-2 positive patient saliva samples collected at the Clemson University testing lab in Upstate South Carolina. Samples were identified as positive using RT-qPCR, and clade information was determined via whole genome sequencing at nearby commercial labs. We also obtained patient-reported information on symptoms and exposures at the time of testing. The lowest Ct values were observed among those infected with Delta (median: 22.61, IQR: 16.72–28.51), followed by Alpha (23.93, 18.36–28.49), Gamma (24.74, 18.84–30.64), and the more historic clade 20G (25.21, 20.50–29.916). There was a statistically significant difference in Ct value between Delta and all other clades (all p.adj<0.01), as well as between Alpha and 20G (p.adj<0.05). Additionally, pre- or asymptomatic patients (n=1093) showed the same statistical differences between Delta and all other clades (all p.adj<0.01); however, symptomatic patients (n=167) did not show any significant differences between clades. Our weekly testing strategy ensures that cases are caught earlier in the infection cycle, often before symptoms are present, reducing this sample size in our population.

**Conclusions:**

COVID-19 variants Alpha and Delta have substantially higher viral loads in saliva compared to more historic clades. This trend is especially observed in individuals who are pre- or asymptomatic, which provides evidence to the high transmissibility and rapid spread of emerging variants. Understanding the viral load of variants spreading within a community can inform public policy and clinical decision making.

## Introduction:

The United States confirmed its first positive SARS-CoV-2 case on January 21, 2020 [1]. As of December 1, 2021, there have been over 265 million cases globally and 48 million in the United States alone. Most recently, clade 21A, classified as the Delta variant, spread rapidly across the globe. On May 29, 2021, the CDC reported that 7.3% of new cases in the U.S.A. were identified as Delta, and 65.4% of cases were clade 20I (Alpha). By August 28, 99.1% of reported cases were Delta [1]. This rapid shift may be attributed to key mutations that increase transmissibility due in part to a higher viral load.

In early 2021, the Alpha variant spread rapidly due to the N501Y mutation in the S protein which enhances its affinity for ACE2. The Delta variant lacks this mutation but carries several mutations within the S protein; specifically, L452R, T478K, and P681R, which confer resistance to monoclonal antibody treatments [2]. The L452R and T478K mutations may also increase transmissibility of the virus by stabilizing the ACE2-RBD complex [2]. Another mutation within the N protein, R203M, increases viral mRNA delivery and expression and facilitates the Delta variant to produce >50-fold more viral particles [3]. These mutations may improve host cell binding affinity, as well as increase viral production, and may contribute to the rapid global spread of this variant.

Most studies of SARS-CoV-2 viral loads used the nasopharyngeal (NP) swab sample collection method [4–6]. While effective at diagnosing infection, there is little evidence that RT-qPCR cycle threshold (Ct) values from these samples are correlated to viral load, and thereby, virus transmissibility. The amount of viral RNA collected by NP swabs may vary in each sample, and therefore, serves as a poor viral load indicator. Alternatively, the viral load in saliva samples has been well correlated with COVID-19 symptoms and transmissibility [7,8]. Low Ct values are associated with high viral load and increased transmissibility [9]. This correlation is primarily due to viral presence in saliva droplets that facilitate spread when infected individuals are in proximity. Saliva has been shown to be an accurate diagnostic tool, yielding comparable Ct values to NP swabs while decreasing both discomfort to patients and risk of transmission to healthcare workers during collection [10].

## Methods:

Ethical review for this study was obtained by the Institutional Review Board of Clemson University. This is a retrospective study on archived deidentified samples and data. The samples and data sets were striped of patient identifiers prior to any SARS-CoV2 sequencing and data analysis. To evaluate the relative viral load of the variants of concern (VOC) found in upstate South Carolina (Alpha, Gamma, and Delta), we compared the Ct values from saliva samples from the SARS-CoV-2 testing lab at Clemson University, which also provides free testing for the surrounding community [11]. University surveillance testing is mandatory for students and employees on a weekly or bi-weekly schedule regardless of vaccination status [12]. The study population includes all university students and employees, as well as members of the surrounding community that tested positive between January and November 2021. Samples were labeled as “symptomatic” if the patient self-reported symptoms at the time of collection, or “exposed” if they reported recent viral exposure. All other samples were considered “surveillance”. Only one positive test was included for each patient; any subsequent tests were excluded from our analysis.

SARS-CoV-2 positive saliva samples were identified using the TigerSaliva RT-qPCR testing method, which targets the N gene [11]. Positive controls made from synthetic RNA at 200 viral copies/μL resulted in a Ct value of approximately 24. It was also determined that a Ct of 33 was equivalent to 1 viral copy/μL, and as such any samples with a Ct lower than 33 were considered positive. Samples were run in duplicate, and the average Ct value from both replicates was used for this analysis.

Heat treated saliva samples were commercially sequenced (Premier Medical Sciences, Greenville, SC; Labcorp, Durham, NC) using the ARTIC protocol. Briefly, RNA was extracted from saliva samples via MagBind Viral RNA Kit (Omega Biotek, Norcross, GA) and recovered SARS-CoV-2 RNA quantity was assessed via Logix Smart COVID-19 assay (Co-Diagnostics, Salt Lake City, UT). Samples with sufficient RNA quantity were sequenced on the Illumina platform using NovaSeq 6000 or NextSeq500/550 flow cell. Sequences were assembled and analyzed using nf-core/viralrecon v.2.2 [13]. Sequence data was uploaded to SC DHEC, GenBank, and GISAID (see Supplementary Data). Some samples had ambiguous regions that prevented database uploads, but all had sufficient information to confidently assign clade by Pangolin and Nexclade.

Ct values among VOCs were compared: 20I (Alpha), 21A (Delta), 20G, and 20J (Gamma, V3) [14]. Due to low prevalence in the Upstate SC community, 20H (Beta) samples (n=8) were excluded from analysis. To maintain phylogenetic independence, we only compare Ct values for variants at branch tips within the NextClade phylogeny [15].

## Results and Discussion:

**Fig 1.**
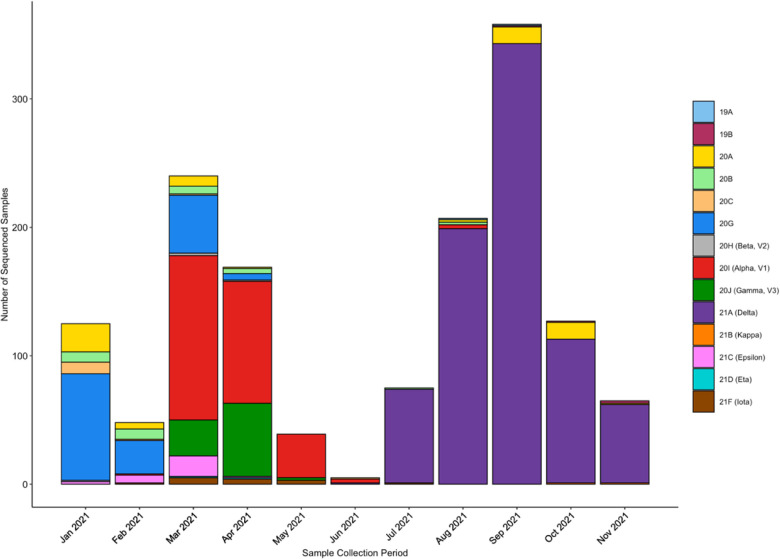
Clade composition of samples run in the REDDI Lab from January to December 2021. Clade determination was made via whole genome sequencing. There were few positive samples between May and June 2021 due to the university summer break.

We first determined the clade composition in our community from the sequenced positive samples between January and December 2021 (Fig 1). From January to July, we sequenced all positive samples stored from the lab. Due to the increase in positive samples during the Delta surge, we sequenced a statistical sampling of positives (approximately 15%) to ensure accurate coverage of our community demographics.

**Figure 2:**
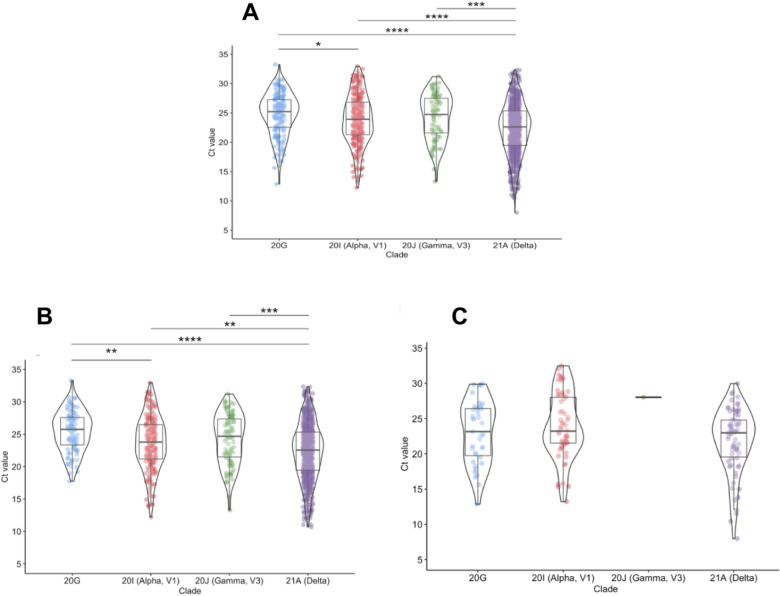
N1 Ct values of common clades in saliva. We analyzed the Ct values from a total of 1297 SARS-CoV-2 positive saliva samples, using the N gene target. **2A: Comparison of all samples.** Delta (n=787) showed a statistically significant difference in Ct value when compared to 20G (n=159), Alpha (n=258), and Gamma (n=87). **2B: Comparison of surveillance samples.** When only surveillance samples were considered, the same trends were observed, showing a significant difference between Delta (n=691) and all other clades (20G: n=95, Alpha: n=181, Gamma: n=86). Both groups also showed a significant difference when comparing Alpha and 20G. **2C. Comparison of symptomatic samples.** There were no significant differences in Ct values observed among symptomatic samples for Delta (n=70), Alpha (n=58), Gamma (n=1), and 20G (n = 39). *p.adj<0.05, **p.adj<0.01, ***p.adj<0.001, ****p.adj<0.0001.

To compare Ct values, and thereby infectious potential, statistical analyses were performed in an R environment using Kruskal-Wallis test followed by Dunn’s test of multiple comparisons (Fig 2). SARS-CoV-2 positive samples showed a significant difference between Delta (median: 22.61, IQR: 16.72–28.51) and all other clades [Alpha: 23.93 (18.36–28.49), Gamma: 24.74 (18.84–30.64), 20G: 25.21 (20.50–29.916)]. When only surveillance samples were considered (Fig 2B), the same trend was observed with Delta (median: 22.56, IQR: 16.67–28.45) having a significantly lower median Ct from other clades [Alpha: 23.81 (18.51–29.11), Gamma: 24.69 (18.84–30.54), 20G: 25.75 (21.53–29.98)]. Additionally, both groups showed a significant difference in Ct values between Alpha and 20G.

When analyzing only symptomatic samples, we found no statistically significant difference in Ct values amongst the clades (Fig 2C). The benefit of Clemson University’s surveillance strategy is that infections are caught early, often before symptoms are present, which decreases the number of symptomatic samples in our population. While there are significant differences in viral loads between the VOC clades and 20G in pre-symptomatic and asymptomatic patients at the time of initial diagnosis, this trend is not necessarily maintained as the disease progresses. This may explain the discordant results in the literature; studies which primarily focused on tests from COVID-19 hospitalized patients did not observe differences in viral loads among the clades [6], whereas studies that included tests from earlier stage diagnoses observed significant differences in viral loads, particularly for Delta [4,5].

**Fig 3:**
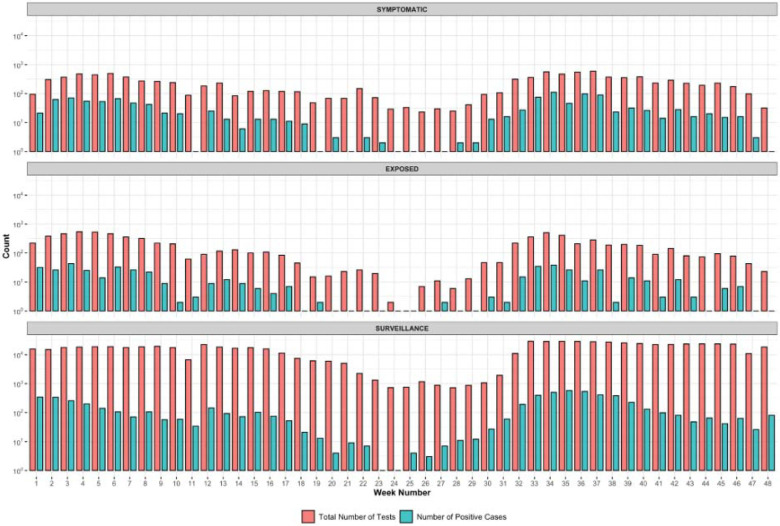
Number of tests and positive tests per category, by week. Note that the y-axis is on a log10 scale. Samples are labeled “symptomatic” if the patient reports symptoms at the time of testing, or labeled “exposed” if they report exposure to a positive patient. Surveillance samples represent the rest of the samples collected. The lower case load during week 11 is due to the university’s spring break, and weeks 18–29 account for summer break.

Additionally, patients that report symptoms are much more likely to test positive compared to non-symptomatic patients (Fig 3). From January to November 2021, the average positivity rate for symptomatic samples was 12.71% and for surveillance samples was 0.98%. During the surge in cases due to the Alpha variant in March 2021, samples from patients at the community site who reported exposure were much more likely to be positive for SARS-CoV-2 when compared to non-exposed (8.8% vs 1.7%). However, after the emergence of Delta, the test positivity rate was 10% in both groups. This is likely due to the overwhelming presence of Delta within our community and the extremely high viral load, likely ensuring that everyone had some level of exposure.

Due to a non-normal data distribution, we performed Kruskal-Wallis test for stochastic dominance. However, it has been suggested that ANOVA is robust to slight non-normality [16]. Reanalyzing the data with Welch’s ANOVA, we observed similar results (SFig 1) and determined there was approximately an 8-fold difference in viral load between Delta and 20G, which is consistent with other studies using NP swabs from initial diagnostic samples [4,5]. Our results highlight the significant difference in Ct values between Delta samples and other VOCs.

## Conclusion:

Overall, our study showcases the increased viral load of the Delta variant and provides evidence for its rapid global spread. A major benefit to saliva-based testing is the ease of testing; people are more inclined to test frequently. Specifically, our data show that the Delta VOC has a higher viral load in saliva even in healthy, young individuals who are pre- or asymptomatic. These individuals are not often captured by other studies as they are not likely to seek out testing; however, they are known to contribute to the rapid spread of COVID-19 [17]. High infectivity of new variants necessitates accurate surveillance. It is expected that future dominant strains, like the newly emerging Omicron, will have viral loads comparable to or greater than Delta to achieve a competitive advantage.

## Supplementary Material

Supplement 1 SFile 1: Accession numbers for sequenced samples uploaded to SCDHEC, GenBank, and GISAID.

Supplement 2 **SFig 1: Analysis of Ct values using Welch’s ANOVA test. 1A: Comparison of all samples**. We observed a statistically significant difference between Delta and all other clades, including an 8-fold difference in viral load when compared to 20G**. 1B: Comparison of only surveillance samples.** The same difference in median Ct was observed between Delta and all other clades. Additionally, surveillance samples showed a statistical difference between Alpha and 20G. *p.adj<0.05, **p.adj<0.01, ***p.adj<0.001, ****p.adj<0.0001

Supplement 3

Supplement 4

## Data Availability

All relevant data are within the manuscript and its Supporting Information files. Data analysis scripts can be found at https://github.com/CUGBF/SARS-CoV-2_Ct-vs-Clade.git
