# Supplementary material for: SARS-CoV-2 variants of concern Alpha and Delta show increased viral load in saliva
Source: medRxiv. 2022 Mar 24:2022.02.10.22270797. Originally published 2022 Feb 15. Preprint. [Version 2] doi: 10.1101/2022.02.10.22270797 (PMC8863157; doi:10.1101/2022.02.10.22270797)
Supplement: 1 [file NIHPP2022.02.10.22270797V2-supplement-1.pdf]

medRxiv preprint doi: https://doi.org/10.1101/2022.02.10.22270797; this version posted March 24, 2022. The copyright holder for this preprint (which was not certified by peer review) is the author/funder, who has granted medRxiv a license to display the preprint in perpetuity.
It is made available under a CC-BY 4.0 International license .

Supporting Information:

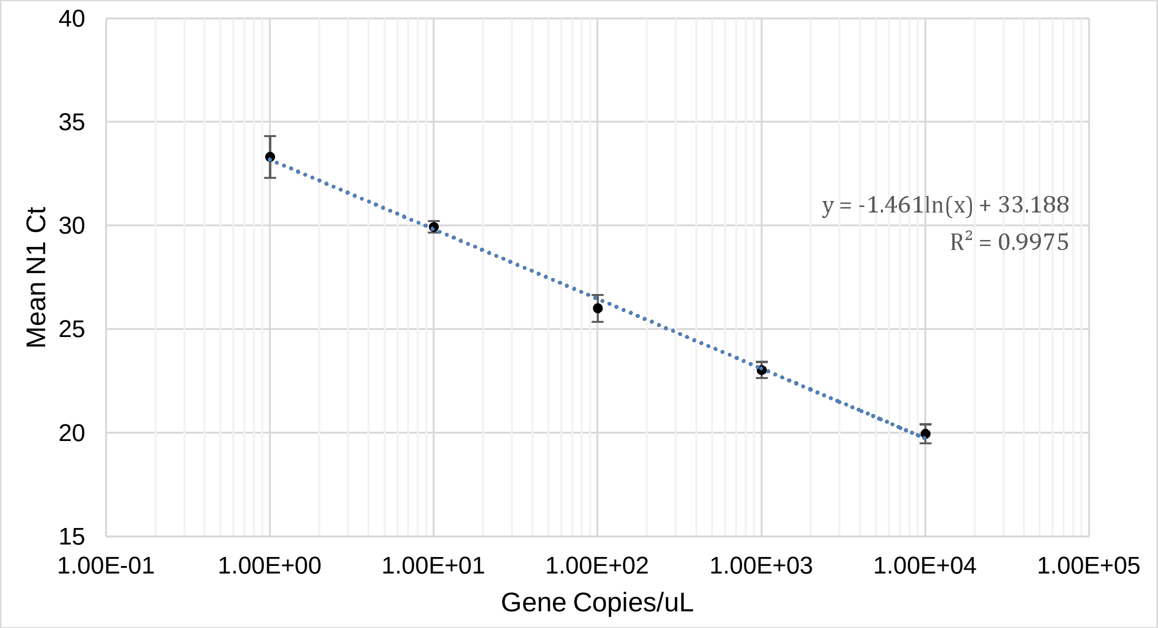

SFig 1: Standard curve for TigerSaliva RT-qPCR assay for N1 detection in synthetic controls. The standard curve was plotted with standard deviations to determine the range of accurate detection using this primer/probe combination. The mean Ct values (n=4) obtained from serial dilutions were plotted against estimated quantify of synthetic RNA in 10µL of RT-qPCR reaction.

medRxiv preprint doi: https://doi.org/10.1101/2022.02.10.22270797; this version posted March 24, 2022. The copyright holder for this preprint
(which was not certified by peer review) is the author/funder, who has granted medRxiv a license to display the preprint in perpetuity.
It is made available under a CC-BY 4.0 International license .

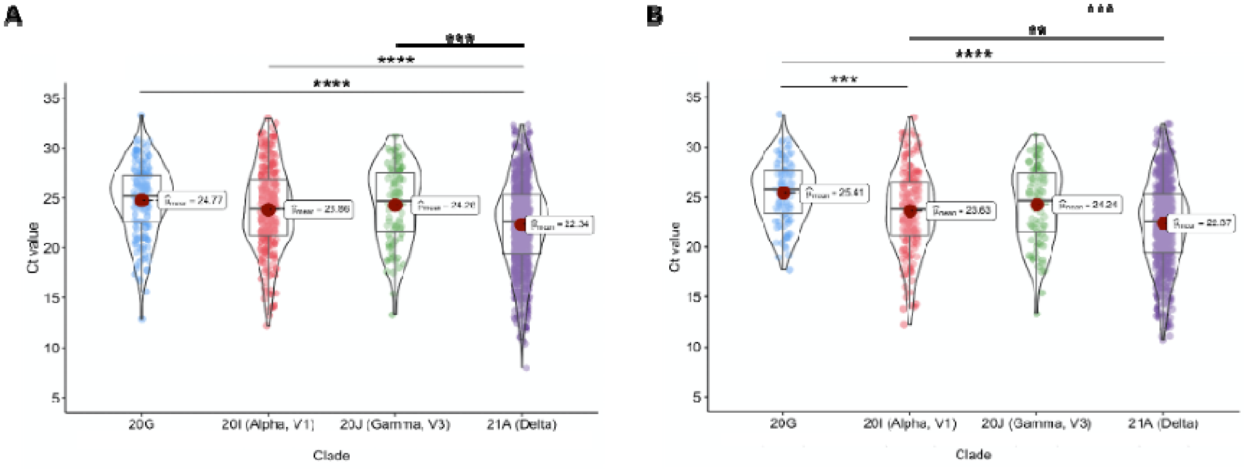

SFig 2: Analysis of Ct values using Welch's ANOVA test. 2A: Comparison of all samples. We observed a statistically significant difference between Delta and all other clades, including an 8-fold difference in viral load when compared to 20G**. 2B: Comparison of only surveillance samples.** The same difference in median Ct was observed between Delta and all other clades. Additionally, surveillance samples showed a statistical difference between Alpha and 20G. *p.adj<0.05, **p.adj<0.01, ***p.adj<0.001, ****p.adj<0.0001

SFile 1: Accession numbers for sequenced samples uploaded to SCDHEC, GenBank, and GISAID.

SFile 2: Demographic Analysis.

SFile 3: Data accessibility for Figures 1 and 2.

SFile 4: Data accessibility for Figure 3.
